# Medical physics workforce in the United States

**DOI:** 10.1002/acm2.13762

**Published:** 2023-01-27

**Authors:** Wayne D. Newhauser, Dustin A. Gress, Michael D. Mills, David W. Jordan, Steven G. Sutlief, Melissa C. Martin, Edward Jackson

**Affiliations:** ^1^ Department of Physics & Astronomy Louisiana State University Baton Rouge Louisiana USA; ^2^ Department of Physics Mary Bird Perkins Cancer Center Baton Rouge Louisiana USA; ^3^ American College of Radiology Reston Virginia USA; ^4^ James Graham Brown Cancer Center Louisville Kentucky USA; ^5^ University Hospitals Cleveland Medical Center Cleveland Ohio USA; ^6^ Banner MD Anderson Cancer Center Phoenix Arizona USA; ^7^ Therapy Physics Inc. Gardena California USA; ^8^ University of Wisconsin Madison Wisconsin USA

**Keywords:** medical physics, workforce

## 3.1 INTRODUCTION

Medical physics is an applied science, with subspecialties in radiation therapy, imaging, nuclear medical physics, and medical health physics (radiation protection). Examples of applications heavily dependent on medical physics expertise include the delivery of external‐beam radiotherapy and radioactive‐seed therapy, computed tomography (CT) and magnetic resonance imaging (MRI), and radiation protection of patients, staff, and the general public within the medical environment. In addition, medical physicists play important roles in the discovery, research and development, and translation of new technologies to clinical practice. Predicting the workforce needs for medical physics is challenging because of limited data availability, unpredictable resource allocation processes, and other factors. In‐line with other radiation professions considered in special issue, the domestic medical physics workforce is experiencing a wave of retirements of baby boomers and a chronic decline in support for programs that educate and/or train replacement workers.

### 3.1.1 Definitions of the profession

This section briefly reviews basic definitions, as well as the scope of responsibilities, of medical physicists. There are considerable areas of overlapping responsibility in the professions of medical physics and health physics, both of which have subspecialties described as medical health physics. This section closely follows descriptions from the American Association of Physicists in Medicine (AAPM).^1^

#### 3.1.1.1 Medical physics

Medical physics is a modern branch of physics concerned with the application of the concepts and methods of physics to the diagnosis and treatment of human disease and is allied with health physics. In this role, medical physicists contribute to the effectiveness of radiological imaging procedures by assuring radiation safety and helping to develop improved imaging techniques (e.g., mammography CT, MR, and ultrasound). They contribute to the development of therapeutic techniques (e.g., prostate implants, and stereotactic radiosurgery), collaborate with radiation oncologists to design treatment plans, and monitor equipment and procedures to ensure that cancer patients receive the prescribed dose of radiation to the correct anatomic location.

The term “qualified medical physicist” applies to an individual who is proven competent to independently practice one or more of the subfields of medical physics: therapeutic physics, diagnostic physics, nuclear medical physics, and medical health physics.^2^ Section 3.1.3 details the pathways and requirements needed to achieve the requisite qualifications. Once qualified, the professional responsibilities of medical physicists can be categorized into the following areas: administrative, clinical services, education, informatics, equipment performance evaluations, quality, and safety (aapm.onlinelibrary.wiley.com/doi/full/10.1002/acm2.12469). A survey of medical physicists^3^ reported that medical physicists spend the highest percentage of their time on patient‐specific clinical tasks (49%), followed by quality assurance (22%), administrative tasks (10%), radiation safety (7%), research (6%), and teaching and training (5%).

Overall, the essential responsibility of a radiation therapy physicist within a clinical practice is to assure the safe and effective delivery of radiation in order to achieve a diagnostic or therapeutic result, performing or supervising the technical aspects of procedures necessary to achieve the objective. Incumbent within this responsibility is as follows: protection of the patient and others from potentially harmful or excessive radiation, establishment of adequate protocols to ensure accurate patient dosimetry, the measurement and characterization of radiation; determination of delivered dose, advancement of procedures necessary to ensure image quality; development and direction of quality assurance programs, assistance to other health‐care professionals in optimizing the balance between the beneficial and deleterious effects of radiation, and compliance with applicable federal and state regulations. In addition, a medical physicist's responsibilities may include teaching a wide variety of subjects to various student populations, including graduate students in medical physics and health physics, medical‐technology students in radiotherapy and imaging technologies, students of radiation dosimetry, and medical physics residents. At academic medical centers, medical physicists may also teach medical residents in radiation oncology, diagnostic radiology, and nuclear medicine.

#### 3.1.1.2 Medical health physics

Radiology, nuclear medicine, and radiation therapy departments are found in almost every modern hospital. Each of these departments utilizes radiation sources, and medical health physicists are involved when radiation sources are used to diagnose and treat human diseases, and to ensure proper and safe working conditions for both patients and medical staff. A medical health physicist often serves as the designated radiation safety officer (RSO) for a medical facility (see Chapter 2 on Health Physics for the details of RSO responsibilities).

### 3.1.2 General characteristics of the workforce

The size of the medical physics workforce may be estimated from the number of members in appropriate professional societies. Using such metrics, there are ∼24 000 medical physicists worldwide,^4^ of which just over a third, or 8200, are in the United States.^5^ The AAPM surveys its membership annually and provides descriptive statistics of relevance to the domestic workforce.^5^ According to the 2019 professional survey conducted by the AAPM, based on data from 2565 respondents, 51% and 49% held MS and PhD degrees, respectively. The majority (76%) of medical physicists were engaged in radiation oncology as their primary subspecialty, nearly all (94%) were employed full time, and only 3% were self‐employed consultants. Reflecting a growing emphasis on certification, in 2015, this survey reported that 81% of respondents were certified, with nearly identical proportions of MS and PhDs. In another recent survey of radiation therapy physicists by the American Society for Radiation Oncology and AAPM,^3^ responses indicated that practices are located mostly in urban communities (55%), with 34% and 12% in suburban and rural settings, respectively. Most medical physicists work in a hospital (41%), private practice (34%) or an academic setting (26%), with the average time worked per week being 48 h.

In both general and relative terms, there are key differences in how the subspecialties of medical physics provide clinical work. Therapy clinics require the continuous presence of a therapy physicist, with most working on a full‐time and continuous basis at a given treatment center, either as an employee or contracting consultant, with the majority of their work being devoted to the care of specific patients and closely related tasks. As some services are provided comparatively infrequently (monthly, quarterly, or annually), the number of diagnostic medical physics employers is somewhat decoupled from the number of providers of imaging services, even though these latter services ultimately drive the need for physics support. The factors that drive workforce needs differ between the subspecialties. For therapy physicists, clinical needs are driven by the number of patients treated or the number of treatments delivered; there are very few patient‐specific needs in diagnostic imaging and nuclear medicine. The workload for quality assurance and radiation safety is commensurate with the number and types of therapeutic and diagnostic devices in each facility and needs are irrespective of the subspecialty.

### 3.1.3 Education and training pathways

Currently, the pathways to becoming a qualified medical physicist are well defined and standardized, beginning with the completion of an undergraduate degree in physics (or a related field with the equivalent of a minor in physics) and concluding with entry‐level employment in the profession. The typical pathway involves participation in a Commission on Accreditation of Medical Physics Education Programs (CAMPEP)‐accredited education program (master of science or doctoral degrees, or a postdoctoral training certificate), followed by a 2‐year medical physics residency training program. In recent years, a few programs have established professional doctorate degree programs (Doctorate in Medical Physics or DMP). Although the DMP pathway is defined, it has also led to some concerns regarding economics and perceptions.^6^ It is worth noting that pathways into nonclinical medical physics careers (e.g., into industry or government) also are well defined, but less standardized, and there is growing interest in such careers, notably in the medical device and related industries. As a result, the AAPM and the Society of Directors of Academic Medical Physics Programs (SDAMPP) are expanding activities to promote and facilitate nonclinical career options.^7^

#### 3.1.3.1 Organizations involved in education

Several professional societies play important roles in medical physics education. In the United States, these include the AAPM, the American Board of Radiology (ABR), the CAMPEP, the American Board of Medical Physics (ABMP), and the SDAMPP. The roles and relationships of these organizations are discussed in greater detail elsewhere (Section 3.1.4.1). In light of the “complexity of interests and areas of potential overlap,” the AAPM, CAMPEP, ABR, and SDAMPP have articulated consensus guidelines to clarify their respective roles in medical physics education, with particular relevance to workforce supply.^8^

#### 3.1.3.2 Undergraduate education

Currently, in the United States, medical physicists typically earn an undergraduate degree in physics, engineering, or other physical science, with several universities offering a BS in medical physics or a concentration in medical physics as an optional component of a traditional BS degree. Although medical physicists in the United States must have at least the equivalent of a minor in physics with their baccalaureate degree, the determination of “equivalence” has some latitude.^9^ Undergraduates may be admitted to graduate programs in medical physics with limited “deficiencies” in required subjects (e.g., human anatomy and physiology), with the assumption they will attain the required knowledge during their graduate studies. Ultimately, however, the adequacy of undergraduate education is assessed by graduate programs in medical physics, the organizations that accredit medical physics programs, organizations that certify and license medical physicists, and employers.

#### 3.1.3.3 Graduate education

Currently all pathways to becoming a medical physicist involve graduate education, with academic medical physics programs offering master of science, doctor of philosophy, or professional doctorate degrees. In the United States, academic medical physics programs are numerous and geographically well distributed, offering a range of degrees and opportunities for supervised research and clinical training. In general, most graduate programs conform to standardized recommendations on curricula. Those programs with CAMPEP accreditation provide education required by regulatory agencies for some positions (see Section 3.1.3.7) and by residency programs for admission to accredited training fellowships.

The number of graduate training programs in medical physics has been increasing for more than three decades, with the number of CAMPEP‐accredited graduate degree programs increasing from 2 in 1988 to 54 by 2019.^10^ Despite this overall growth, student demand for medical physics graduate education far exceeds supply; in 2019, there were a total of 1914 applications, with only 677 offers of admission and 284 matriculations.^10^ Most graduates from academic programs apply for admission to a medical physics residency training program. According to CAMPEP, 74% of MS graduates who applied to a residency program were accepted, 98% of PhD graduates who applied were accepted, and 67% of postdoctoral certificate graduates were accepted into a residency program. Market forces govern the admissions of graduate and residency programs.

#### 3.1.3.4 Postgraduate training

Options for additional training after completion of the terminal graduate degree include enrollment in a residency training program and/or a postdoctoral training program.

##### Residency training

Since 2014, eligibility to sit for an ABR certification exam requires graduation from a CAMPEP‐accredited residency program; as a consequence, the capacity of medical physics residency training programs in the United States has grown rapidly in recent years. Furthermore, for non‐DMPs, completing a residency is a necessary step in the pathway to becoming a qualified medical physicist. Currently, there are over 110 CAMPEP‐accredited residency programs in North America, including 94 in therapy physics and 21 in imaging physics. Residency training programs typically include at least 2 years of clinical training; the AAPM has recommended curricula for medical physics residency programs^11^ and standards for the accreditation of residency programs have been established by CAMPEP.^9^ However, the number of residency training openings is fewer than the number of applicants; consequently, the application process is extremely competitive, and the number of available residency positions is, at present, the predominant factor limiting the supply of new workers.

##### Postdoctoral training

The supply of new medical physicists is supplemented by postdoctoral research fellows. Typically, postdoctoral fellows hold PhD degrees in medical physics, but also in physics, nuclear engineering, or other related physical sciences. For many of these trainees, that is, those with degrees other than medical physics, the postdoctoral fellowship is an alternate pathway to the more common pathway of earning an accredited degree in medical physics; however, the variability in training programs with direct relevance to clinical medical physics complicates the assessment of professional qualifications.

#### 3.1.3.5 Alternate pathways

A formalized alternate pathway into clinical medical physics, that is, a postdoctoral certificate program, was introduced in 2011 and involved a recommended curriculum^12^; this included some of the core classroom courses from the recommended MS or PhD degree, with standards for accreditation.^13^, ^14^ In 2014, there were 17 accredited postdoctoral certificate training programs, who collectively had 49 trainees enrolled and 21 graduates.^15^ As the certificate programs are a relatively new development, there are insufficient available statistical data to discern trends that will impact the supply of medical physics workers, especially as the number of postdoctoral fellows at present is relatively small (7% of graduates from medical physics degree programs). Nonetheless, the certificate program has features that may increase the elasticity of supply, including the speed of training and the disciplinary diversity of its graduates.

#### 3.1.3.6 On‐the‐job training

Typically, eligibility for board certification in clinical medical physics requires residency training, followed by a period working under the supervision of a board‐certified medical physicist. Additional formal on‐the‐job clinical training (beyond the minimum requirements described before) may be required to become an authorized medical physicist under the Nuclear Regulatory Commission (NRC) or may be required to obtain institutional credentials to perform some special procedures. Physicists who are certified by ABR in a specialty of medical physics may obtain certification in an additional specialty after having completed 1 year of on‐the job training.

#### 3.1.3.7 Professional certification and licensure

Requirements for professional certification and licensure apply to the overall practice of clinical medical physics but vary among different states and employment settings. The requirements also differ among the subspecialties of diagnostic physics, nuclear medical physics, and therapy physics. The primary mechanism for certification of clinical medical physicists in the United States is through the ABR, which, following completion of residency, offers certification in all three subspecialties. ABR certification in the applicable subfield is recognized as sufficient qualification for issuance of a license, certificate, registration, accreditation, or permit in all regulatory jurisdictions that regulate the practices of diagnostic and therapeutic medical physics. It is also recognized by national accreditation programs offered by American College of Radiology (ACR), Intersocietal Accreditation Commission, Joint Commission, etc. In some jurisdictions (e.g., municipalities, states), it is the minimum qualification needed to practice, with no alternative pathways available. The ABMP offers certification in medical health physics. Those physicists serving as a RSO in an institution requiring a radioactive material (RAM) license must meet specific qualification requirements, be individually approved by the NRC or agreement state, and listed by name on each RAM license. There are a number of training and experience pathways to qualify as an RSO (10 Code of Federal Regulations § 35.50) and medical physicists are not the only individuals qualified to serve in this capacity. ABR certification in any subfield of medical physics can be used with relevant work experience to qualify an individual as an RSO. Certification in nuclear medicine physics and instrumentation by The American Board of Science in Nuclear Medicine (ABSNM) is recognized by the NRC for RSO qualification, by the four states requiring licensure in medical nuclear physics, and by the ACR and Joint Commission accreditation programs for qualification of physicists providing nuclear medicine and PET physics support. The ABSNM certifications in Nuclear Medicine Physics and Instrumentation, and Radiation Protection are also recognized by the NRC for RSO eligibility purposes, as is ABMP certification in medical health physics.

##### Diagnostic and therapeutic medical physics certification

There are no certification pathways besides the ABR available to new medical physicists seeking initial certification in therapeutic medical physics. Medical physicists who were previously certified in diagnostic or therapeutic medical physics by the ABMP are recognized as holding ABR‐equivalent certification under an agreement between the ABR and ABMP. ABMP certifies individuals in MRI physics

##### Nuclear medicine medical physics certification

Presently, four states define and regulate the practice of nuclear medicine physics and prohibit unlicensed individuals from performing such duties. Nuclear medicine physics duties may or may not require the medical physicist to hold, or be recognized on, a RAM license.

#### 3.1.3.8 Continuing education

In general, medical physicists maintain and acquire new knowledge through a variety of continuing education (CE) activities. These are required to maintain professional certification, licensure, and other credentials. Although requirements vary by jurisdiction, the reader is referred to the CE requirements of the ABR (2016) and ABMP (https://abmpexam.com/recertification/). For those board‐certified medical physicists that are grandfathered into limited CE requirements, the ACR Accreditation Program Requirements for Medical Physicists and MR Scientists may be considered a practical alternative standard for CE.^16^

### 3.1.4 Professional aspects of relevance to workforce supply

#### 3.1.4.1 Professional organizations

There are myriad organizations that are centrally or peripherally involved with medical physics. These may be classified by the geographic locations of the participating memberships. For the interested reader, links to lists of organizations are available online.^17^

##### International organizations

The International Organization for Medical Physics claims a membership of over 80 national medical physics organizations. It has the mission of advancing medical physics practice worldwide through the dissemination of scientific and technical information, fostering the educational and professional development of medical physics and promoting the highest quality medical services for patients. It works together with several international organizations, including the International Atomic Energy Agency, whose scope includes significant activities of relevance to medical physics (e.g., to increase the safety of radiation therapy^18^).

##### Domestic organizations

The AAPM is the primary society of most domestic medical physicists and has the largest membership (about 9000). The society is active in scientific and professional matters, including workforce, training, and education topics. Its primary goal is the identification and implementation of improvements in patient safety following the medical use of radiation in imaging and radiation therapy. Furthermore, through its government affairs activities, AAPM has established a cooperative working relationship with numerous government bodies and organizations, including the Congress, federal and state agencies, such as the NRC, Food and Drug Administration and Environmental Protection Agency, as well as related professional societies. Other professional societies cover broader occupational categories than medical physics but often overlap with medical physics interests through standards recommendations, publications, and advocacy.

#### 3.1.4.2 Interdependencies with other radiation professions

Medical physics work is typically interdisciplinary, most especially through interactive instruction with other radiation professionals, including physicians, radiobiologists, and health physicists. Several medical physics and health physics graduate degree programs cross list or share courses taken by students of either discipline. These include topics such as shielding, radiation protection, radiation interactions, radiation instrumentation, dosimetry, and radiation biology.

### 3.1.5 Current status and future outlook

The majority (∼75%) of medical physicists specialize in radiation therapy. Therefore, workforce issues are dominated by needs associated with radiation therapy, which is mainly used to treat cancer. Indeed, radiation is used to treat about half of all cancer patients^19^ and various staffing models have been used to calculate the workforce demands for a radiotherapy clinic.^20^, ^21^ Currently, approximately one medical physicist is needed for every 300 radiotherapy patients treated annually, although this number can vary considerably depending on a range of factors, including the model selected, the practice setting, and also will show variability over time, contingent on changes in practice and technology. The absolute cancer incidence in the United States is increasing by ∼2% annually due to growth and aging of the population. Calculations using the domestic demographic workforce data from 2012 from Chen et al.^3^ suggest that retirement from the medical physics workforce comprises a 2.2% drain on supply per year.

However, future attrition is expected to accelerate as the cohort of baby boomers retires. According to Mills et al.,^22^ the rate of retirement between 2010 and 2020 was approximately triple that seen between 1990 and 2000. Furthermore, as described in Section 3.1.3.4, residency training capacity has been lagging the output of graduates from degree and certificate programs, creating a bottleneck in the supply line of entry‐level medical physicists and, consequently, a glut of graduates who have been unable to gain admission to residency programs. This issue has garnered attention and controversy regarding if and how to regulate admissions.^23^ Another factor affecting the supply and capabilities of workers is the coupling of training programs to federal support for higher education and research. In particular, the number, size, and capacity of graduate education programs depend, to varying degrees, on federal funding, which has been declining nationally for both higher education^24^ and research.^25^, ^26^, ^27^

The future of the workforce has received considerable attention in the literature; historical, and current data are available, and various models are available that are of relevance to the workforce. Many of the governing factors are reasonably well known with small uncertainties (e.g., cancer incidence and the approximate capacities of training programs). Clearly, imponderables abound, such as the impact of health‐care (economic) reform, utilization of radiation in medicine, efficiency of medical physics procedures, and the shape of the looming wave of retirements. For these reasons, trends in supply and demand are difficult to predict for more than a few years into the future.

### 3.1.6 Summary and recommendations

Medical physics is a well‐defined, established, and mature profession. There is an expansive body of literature regarding the present‐day workforce and its current status is known in detail and with certainty; at present, the size of the domestic medical workforce appears in balance with the nation's needs. There are, however, too few residency training positions available to accommodate the number of current graduates from medical physics degree programs. Furthermore, the profession and its workforce has undergone rapid changes, driven by advances in science and medicine, health‐care reform, and by an increasing emphasis on professional aspects of medical physics. Unfortunately, current models and projections for staffing levels and needs are highly uncertain and cannot reliably forecast the situation beyond a few years from the present. Consequently, it is not clear whether the workforce will be adequate in the near future. Vigilant surveillance and long‐range planning will be essential to ensure the future adequacy of the nation's professional medical physics workforce. Recommendations to mitigate against the risks of future workforce inadequacies include improving flexibility in training opportunities, the collection and analysis of additional workforce data, dissemination and discussion of findings, policy assessments, and risk and crisis management activities, as appropriate.

The following recommendations are consensus expert opinions on actions needed to ensure that the medical physics profession will be able to meet the nation's future needs. The authors intentionally declined to recommend detailed methods, timelines, responsibilities of individual organizations, and funding sources. These complex subjects are outside the scope of this work.

**The authors recommend the following items to ensure the future adequacy of the nation's professional medical physics workforce**.

1. The medical physics profession should continue its annual surveillance and analysis of data regarding the status of the medical physics workforce by cognizant professional societies, including monitoring of recent or possible future changes in policy, law, regulations, and standards of relevance.
2. Based on consistent and adequate survey from annual surveys, longitudinal analyses should be performed to better understand long‐term trends.
3. Coordinate and collaborate with survey efforts of other radiation professions in order to enhance the direct comparability of survey data, for example, especially with the closely related profession of health physics, which also has the subspecialty called medical health physics.
4. Future projections should be modeled to provide evidence to inform decision making by universities, employers, future workers (e.g., students), and other stakeholders.
5. Professional organizations and regulatory agencies of the federal government should monitor policies and regulations of relevance to the adequacy of the future workforce. These should be disseminated to stakeholders to inform decision making.
6. Medical physics professional organizations should proactively work to mitigate the risk that workforce shortages cause future societal needs to go unmet. Currently this is of enhanced importance because of the imminent wave of retirement of older workers.

The previous recommendations differ somewhat from the recommendations in a statement by the National Council on Radiation Protection (NCRP).^28^ The earlier NCRP recommendations are broader, pertain to all of the radiation professions, yet are applicable to the profession of medical physics. Additional discussion and recommendations of relevance were reported by Coleman et al.^29^ and Dynlacht et al.^30^

## AUTHOR CONTRIBUTION

All the authors listed have contributed directly to the intellectual content of the manuscript.

## CONFLICT OF INTEREST

No conflict of interest.

## Data Availability

Data sharing is not applicable to this article as no additional new data were created or analyzed in this study.
